# Local circuit model of the subthalamo-pallidal network for the generation of parkinsonian oscillations

**DOI:** 10.1186/1471-2202-15-S1-P168

**Published:** 2014-07-21

**Authors:** Osamu Shouno, Kenji Doya

**Affiliations:** 1 Okinawa Institute of Science and Technology Graduate University, Tancha, Onna-son, Okinawa 904-0495, Japan; 2 Honda Research Institute Japan Co., Ltd., Honcho, Wako, Saitama 351-0188, Japan

## 

Parkinson’s disease is psychomotor dysfunctions caused by degeneration of nigral dopaminegic neurons. Increasing evidence suggests that abnormally synchronized oscillatory activity in the basal ganglia contributes to the expression of parkinsonian motor symptoms. Experimental works demonstrated that neurons in the subthalamic nucleus (STN) and the internal (GPi) and external segments of the globus pallidus (GPe) of dopamine-depleted animals exhibit enhanced oscillatory burst discharges in 8-15 Hz, and suggest that interactions between STN and GPe generate these pathological oscillations [1]. However, the biophysical and neuronal circuitry mechanisms for the generation of these abnormal oscillations by the dopamine depletion remain poorly understood. We addressed this problem by computer simulations of spiking neuron models of the local STN-GPe circuitries with the biophysical properties of neurons and synaptic connections [2-4]. These simulations revealed that the connection patterns between STN and GPe neurons, the high maximal conductance of GPe-to-STN synapses, a type of short-term plasticity at the glutamatergic synapses of GPe neurons, and the reduced baseline firing activity of GPe neurons were critical for generation of oscillatory burst discharges in 10-15 Hz. These latter two conditions were consistent with experimental evidence [4-6]. Characterization of the mechanisms underlying these oscillations suggest that a moderate level of STN post-inhibitory rebound excitation shapes the 10-15 Hz oscillatory bursts, and that strengthened GPe-STN connectivity and reduced autonomous firing of GPe neurons are critical for the induction of the STN rebound excitation. These results suggest that the physiological and structural changes in the local subthalamo-pallidal circuits caused by the dopamine depletion underlie the induction of the 10-15 Hz parkinsonian oscillations in the STN.

## References

[B1] Tachibana Y Iwamuro H Kita H Takada M Nambu A Subthalami-pallidal interactions underlying parkinsonian neuronal oscillations in the primate basal ganglia Eur J Neurosci 2011 15 1470 1484 10.1111/j.1460-9568.2011.07865.x 22034978

[B2] Otsuka T Abe T Tsukagawa T Song WJ Conductance-based model of the voltage-dependent generation of a plateau potential in subthalamic neurons J Neurophysiol 2004 15 255 264 10.1152/jn.00508.2003 15212440

[B3] Hanson JE Jaeger D Short-Term Plasticity Shapes the Response to Simulated Normal and Parkinsonian Input Patterns in the Globus Pallidus J Neurosci 2002 15 5164 5172 12077211 10.1523/JNEUROSCI.22-12-05164.2002 PMC6757739

[B4] Atherton JF Menard A Urbain N Bevan MD Short-term Depression of External Globus Pallidus- Subthalamic Nucleus Synaptic Transmission and Implications for Patterning Subthalamic Activity J Neurosci 2013 15 7130 7144 10.1523/JNEUROSCI.3576-12.2013 23616523 PMC3678728

[B5] Fan KY Baufreton J Surmeier DJ Chan CS Bevan MD Proliferation of External Globus Pallidus-Subthalamic Nucleus Synapses following Degeneration of Midbrain Dopamine Neurons J Neurosci 2012 15 13718 13728 10.1523/JNEUROSCI.5750-11.2012 23035084 PMC3475197

[B6] Chan CS Glajch KE Gertler TS Guzman JN Mercer JN Lewis AS Goldberg AB Tkatch T Shigemoto R Fleming SM HCN channelopathy in external globus pallidus neurons in models of Parkinson’s disease Nat Neurosci 2011 15 85 92 10.1038/nn.2692 21076425 PMC3058391

